# Association between Klotho levels in cerebrospinal fluid and choroid plexus enlargement in neurodegeneration

**DOI:** 10.3389/fnagi.2025.1688996

**Published:** 2025-11-27

**Authors:** Luca Sacchi, Marina Arcaro, Giorgio Bocca, Lia Schmid, Tiziana Carandini, Laura Ghezzi, Manuela Pintus, Anna Margherita Pietroboni, Chiara Fenoglio, Maria Serpente, Giorgio Conte, Fabio Triulzi, Raffaella Lanzarotti, Claudia Dolci, Daniela Galimberti, Andrea Arighi

**Affiliations:** 1 Department of Biomedical, Surgical and Dental Sciences, University of Milan, Milan, Italy; 2 Fondazione IRCCS Ca' Granda, Ospedale Maggiore Policlinico, Milan, Italy; 3 Department of Biomedical Sciences for Health, University of Milan, Milan, Italy; 4 Faculty of Mathematics and Computer Science, University of Heidelberg, Heidelberg, Germany; 5 PHuSe Lab, Department of Computer Science, University of Milan, Milan, Italy; 6 Department of Pathophysiology and Transplantation, University of Milan, Milan, Italy

**Keywords:** glymphatic system, aquaporin-4, choroid plexus, Klotho, Alzheimer’s disease

## Abstract

**Background:**

Klotho is a longevity-associated protein found in membrane-bound and secreted forms, with the latter detectable in blood and cerebrospinal fluid (CSF). Circulating Klotho mainly originates from the kidney, while the choroid plexus (CP) secretes it into the CSF. CP dysfunction is associated with reduced Klotho expression and neurodegeneration and may result in CP enlargement on magnetic resonance imaging (MRI). In this preliminary study, we investigated Klotho levels in neurodegenerative patients and their association with CP enlargement.

**Materials and methods:**

We retrospectively analyzed 40 patients from the IRCCS Ca′ Granda Ospedale Policlinico, Milan, including 32 neurodegenerative patients (Deg) and 8 cognitively normal controls (NonDeg). CSF and serum Klotho levels were measured using an ELISA kit. KL-VS and apolipoprotein E (APOE) genotyping were performed. CP volumes were segmented using ITK-SNAP and normalized to total intracranial volume (TIV), resulting in a measure known as the CP volume fraction (CPVF). A multivariate linear regression analysis was conducted, adjusting for diagnostic group, age, sex, APOEε4, CPVF, and gray matter volume fraction (GMVF).

**Results:**

CSF Klotho levels were significantly lower in Deg patients (mean = 729 pg./mL, SD = 364) compared to NonDeg individuals (mean = 1,077 pg./mL, SD = 220) (*t* = 3.44, *p* = 0.003). Higher CPVF (*β* = −0.34, 95% CI [−0.64, −0.05], *p* = 0.023) was independently associated with lower CSF Klotho levels.

**Conclusion:**

In this preliminary study, we observed a strong association between CSF Klotho levels and CP enlargement. Reduced CSF Klotho levels, due to CP dysfunction, may contribute to neurodegeneration. If confirmed in larger cohorts, this association suggests that CSF Klotho may serve as a biomarker for CP enlargement, possibly reflecting its underlying dysfunction.

## Highlights

- Klotho levels in cerebrospinal fluid are significantly lower in neurodegenerative patients compared to older controls
- Lower Klotho levels in cerebrospinal fluid are associated with choroid plexus enlargement on magnetic resonance imaging
- Klotho levels in cerebrospinal fluid could serve as a marker of choroid plexus enlargement and dysfunction in neurodegenerative conditions

## 1 Introduction

The choroid plexus (CP), located within the brain ventricular system, is the primary site of cerebrospinal fluid (CSF) production, forms the blood–CSF barrier, and plays a key role in peripheral–central immune surveillance (Zhu et al., 2018). Furthermore, the CP supports neuronal functioning by producing a large variety of neurotrophic factors during embryonic development and adulthood (Sadanandan et al., 2024), including Klotho.

Klotho is a transmembrane protein primarily expressed by the kidneys, parathyroid glands, and CP (Semba et al., 2014). The extracellular domain of the Klotho protein undergoes shedding, resulting in two forms: membrane-bound Klotho and secreted Klotho (Massó et al., 2015; Mengel-From et al., 2016). The latter acts on distal organs as an endocrine substance, exerting pleiotropic but largely unknown effects (Kuro-o, 2019). The level of secreted Klotho is increased in heterozygotes for a common haplotype of six missense variants in the human *klotho* gene, termed KL-VS, while it is reduced in KL-VS homozygotes compared to major allele homozygotes (Yokoyama et al., 2017; Chen et al., 2023).

The *klotho* gene was originally discovered in a mutant mouse strain that exhibited features resembling premature human aging and a shortened lifespan (Kuro-o et al., 1997). Consistently, Klotho levels in humans decline with age and age-related diseases, such as chronic kidney disease (Kuro-o, 2019; Mattinzoli et al., 2023), and reductions in Klotho levels are associated with worse cognition in older people (Shardell et al., 2016; Yokoyama et al., 2017; Kundu et al., 2022; Wu et al., 2023; Ge et al., 2024) and an increased risk of all-cause mortality (Prud’homme et al., 2022).

Beyond its role in protecting against the effects of normal aging, multiple levels of evidence suggest that Klotho reduces the risk of neurodegenerative diseases.

Pre-clinical studies indicate a protective role for Klotho across various disease models (Brobey et al., 2015; Dubal et al., 2015; Sedighi et al., 2019; Zeldich et al., 2019; Zeng et al., 2019; Ho et al., 2020; Zhao et al., 2020). In humans, KL-VS heterozygosity has been associated with a reduced burden of amyloid and tau pathology in Alzheimer’s disease (AD) (Erickson et al., 2019; Belloy et al., 2020, 2021; Neitzel et al., 2021; Ali et al., 2022; Driscoll et al., 2022; Grøntvedt et al., 2022), potentially offsetting the negative effect of apolipoprotein E (APOE)-ε4 carrier status (Erickson et al., 2019; Belloy et al., 2020, 2021; Tank et al., 2021; Chen et al., 2023), one of the most well-established genetic risk factors for AD. Moreover, it has been associated with slower cognitive decline in both AD (Chen et al., 2023) and Parkinson’s Disease (PD) (Zimmermann et al., 2021). In addition, recent findings indicate reduced expression of the *klotho* gene in patients with AD and frontotemporal dementia (FTD) compared to older healthy controls, independent of genotype, suggesting a role for Klotho in shared pathways of neurodegeneration (Sorrentino et al., 2023). Moreover, higher levels of Klotho in CSF are associated with reduced pathological burden and enhanced cognitive function in AD (Semba et al., 2014; Grøntvedt et al., 2022) and PD (Yalcin et al., 2024; Zimmermann et al., 2024). In contrast, findings on peripheral Klotho levels have been less consistent and do not always align with those observed in CSF (Grøntvedt et al., 2022).

CP enlargement, detectable by structural magnetic resonance imaging (MRI), is another recognized feature of aging (Čarna et al., 2023; Hidaka et al., 2024) and neurodegeneration (Choi et al., 2022; Assogna et al., 2023; Jeong et al., 2023; Jiang et al., 2023, 2024). Growing evidence suggests that CP enlargement may reflect underlying CP dysfunction, although this relationship is not yet fully established. In turn, CP dysfunction has been associated with reduced expression of Klotho in choroid epithelial cells (Zhu et al., 2018; Sadanandan et al., 2024).

Against this background, in this preliminary study, we tested the hypothesis that reduced Klotho levels are associated with CP enlargement in neurodegenerative patients compared to older cognitively normal individuals. In addition, we explored the reciprocal relationship between Klotho levels in CSF and serum and their association with KL-VS haplotype, clinical and biological features, and cognitive performance.

## 2 Materials and methods

### 2.1 Study participants

For this study, we retrospectively selected 40 individuals who underwent a comprehensive neurological work-up for suspected dementia at the Neurodegenerative Diseases Unit of the Fondazione IRCCS Ca′ Granda Ospedale Maggiore Policlinico in Milan, Italy.

The eligibility criteria included the following: (1) a diagnosis of either normal cognition or neurodegenerative cognitive decline, specifically amnestic AD or behavioral variant FTD; (2) the availability of a brain MRI scan performed on a 3 T unit (Philips Achieva, dStream, Eindhoven, Netherlands); (3) a banked CSF sample; and (4) a banked blood sample suitable for genetic testing. The participants were categorized into two groups: the neurodegenerative (Deg) group, comprising individuals with a final diagnosis of amnestic AD or behavioral variant FTD, according to current criteria (Rascovsky et al., 2011; Dubois et al., 2021), and the non-neurodegenerative (NonDeg) group, which included cognitively unimpaired older individuals without evidence of neurological disorders or neurodegeneration at work-up or follow-up.

Written informed consent was obtained from all participants or their legal proxies. The study was approved by the local Ethics Committee (Comitato Etico Area 2 Milano, approval N 859_2021, dated 14 September 2021).

### 2.2 Assessment of KL-VS heterozygosity and APOE status

An allelic discrimination assay was conducted in both patients and controls to determine the distribution of the three KL-VS genotypes—wild-type, heterozygote, and homozygote. Genomic DNA was extracted from whole blood, and a TaqMan allelic discrimination assay (Thermo Fisher Scientific) was performed using the QuantStudio 12 K Real-Time System (Applied Biosystems). The analysis targeted two tagging SNPs that define the KL-VS variant: rs9536314 (T/G) for F352V and rs9527025 (G/C) for C370S. In addition, all participants were genotyped for APOE alleles using SNPs rs429358 and rs7412. The included participants were subsequently categorized based on their KL-VS genotype (KL-VShet+ or KL-VShet−) and APOE status (APOEε4 + or APOEε4−, defined by the presence of at least one APOEε4 allele) for further analysis.

### 2.3 Sampling and analysis of CSF and serum

CSF samples were available for all 40 participants, while serum samples suitable for Klotho measurement were available for 26 of them. CSF was collected in 15 mL polypropylene tubes via lumbar puncture at the L3/L4 or L4/L5 interspace between 8:00 and 10:00 a.m., following overnight fasting. After collection, the CSF samples were centrifuged at 2000 rpm for 10 min at 4 °C. Simultaneously, serum samples were collected in sterile polypropylene tubes without anticoagulant and centrifuged at 2,000 × g for 10 min at room temperature.

The processed CSF and serum samples were aliquoted into 0.5 mL polypropylene tubes and immediately frozen at −80 °C for subsequent analysis. Klotho protein levels were quantified in CSF (40/40 samples) and serum (26/40 samples) using commercial ELISA kits (IBL, Japan), following the manufacturer’s instructions. The serum samples were diluted twofold, while the CSF samples were analyzed undiluted. The assay’s detection limit was 6.15 pg./mL, with a measurement range of 93.75–6,000 pg./mL. All readings were performed using a Tecan Multiplate Reader at 450 nm. In addition, creatinine levels were measured at the time of lumbar puncture.

### 2.4 CP segmentation

The CP was manually segmented on 3D T1-weighted images by GB and LiS, a bioengineer and an informatician trained in CP volumetry. Segmentation was performed using the ITK-SNAP software (version 3.8.0).^1^ To account for variability in MRI protocols among the participants, all images were resliced prior to segmentation to match the resolution of the image with the lowest spatial resolution (180 × 240 × 240, isotropic 1 mm voxels). The 1 mm isotropic resolution was chosen to ensure consistent spatial sampling and visualization for manual segmentation across all participants. A visual inspection of all bilateral CP masks was performed (see Supplementary Figure 1 for a representative illustration). Tissue probability maps of gray matter (GM), WM, and CSF were generated using Statistical Parametric Mapping (SPM12, Wellcome Trust Centre for Neuroimaging) for total intracranial volume (TIV) calculation. CP and GM volumes were expressed as the ratio of TIV (CP and GM volumes were normalized by dividing each by the total intracranial volume (TIV)) to account for head size variability.

### 2.5 Statistical analysis

Statistical analyses were conducted using R Studio (version 2024.12.0). Data normality was assessed by visual inspection of histograms and the Shapiro–Wilk test. Initially, univariate analyses were performed using the built-in *t.test* function for group comparisons—which does not assume equal variances and is robust to unequal group sizes—and Pearson’s or Spearman’s correlation analysis, depending on data normality. Multivariate regression models were constructed using forward stepwise selection of variables based on the Akaike information criterion (AIC) to reduce the risk of overfitting while maintaining optimal predictive performance.

The full regression models included diagnostic group, age, sex, CPVF, and GMVF, as well as APOE carrier status and KL-VS genotype—given their potential influence on Klotho levels or function—as covariates. For models predicting mini–mental state examination (MMSE) scores, education was included as an additional covariate. A separate analysis was conducted within the Deg subgroup, excluding diagnostic group from the covariates. Non-normally distributed variables were log-transformed, and MMSE scores were inverted and log-transformed using the formula log(31 − MMSE) to correct for left skew. All variables were standardized prior to model fitting.

Model assumptions, including linearity, homoscedasticity, and normality of residuals, were evaluated using diagnostic tools such as the Shapiro–Wilk tests, Q-Q plots, and residual plots. Statistical significance was defined as a *p*-value of <0.05 (uncorrected).

Moreover, to assess the stability of multivariate models and quantify parameter uncertainty, a bootstrap resampling procedure was applied to the full dataset and the Deg subgroup (1,000 iterations). In each iteration, the forward-selected model was refitted, and variable inclusion frequencies were derived.

## 3 Results

Among the included participants, 32 were classified as Deg patients (F: M 15:17; median age 71, IQR [64, 75.25]), including 19 with AD and 13 with FTD. A total of eight participants were classified as NonDeg cognitively unimpaired participants (F: M 3:5; median age 70.5, IQR [64.5, 74.75]). Demographic and clinical data are summarized in Table 1.

**Table 1:** Demographic and clinical characteristics of the full sample, and separately for the degenerative and non-degenerative groups.

	NonDeg *N* = 8 (6)	Deg *N* = 32 (20)	Total *N* = 40 (26)
Sex (n)			
Female: Male	3:5	15:17	18:22
Education (years)			
Median [Q1, Q3]	8 [8, 13]	13 [8, 13]	12 [8, 13]
Age at CSF (years)			
Median [Q1, Q3]	70.5 [64.5, 74.75]	71 [64, 75.25]	71 [64, 75.25]
MMSE at CSF			
Median [Q1, Q3]	26.5 [24.75, 29]	24 [21.75, 27]	22 [22, 27]
APOE status (n)			
APOEε4 carriers −/+	7:1	19:13	26:14
KL-VS status			
KL-VS heterozygotes −/+	5:3 (3:3)	26:6 (19:1)	31:9 (22:4)

NonDeg, non-degenerative group; Deg, degenerative group; CSF, cerebrospinal fluid; MMSE, Mini-Mental State Examination; APOE, apolipoprotein E. Numbers in parentheses indicate the subgroup of patients for whom serum Klotho concentrations were available.

### 3.1 Univariate analysis

#### 3.1.1 Klotho concentrations and clinical and demographic variables

Klotho levels in CSF and serum were not correlated. Neither CSF nor serum Klotho levels showed significant differences based on sex. CSF Klotho concentrations were not associated with age and creatinine levels, whereas serum Klotho levels exhibited significant negative associations with both age (Spearman’s rho = −0.43, *p* = 0.029) and creatinine levels (rho = −0.45, *p* = 0.029) (Table 2).

**Table 2:** Univariate correlation analysis results.

	Klotho serum		Klotho CSF	
	*r*	*P*-value	*r*	*P*-value
Klotho serum	–	–	0.27^a^	0.18
Klotho CSF	0.27^a^	0.18	–	–
Age at CSF	**−0.43** ^**b**^	**0.03**	0.09^b^	0.58
Creatinine at CSF	**−0.45** ^**a**^	**0.03**	0.11^a^	0.51
MMSE at CSF	0.01^b^	0.96	**0.35** ^**b**^	**0.03**
CPVF	−0.05^b^	0.82	**−0.32** ^**b**^	**0.04**

^a^Pearson’s r. ^b^Spearman’s rho.

Bold values show significant correlations.

#### 3.1.2 Klotho concentrations stratified by KL-VS status

Both CSF (mean = 975 pg./mL, SD = 441 vs. mean = 747 pg./mL, SD = 331) and serum (mean = 907 pg./mL, SD = 241 vs. mean = 732 pg./mL, SD = 310) Klotho levels were consistently higher in the KL-VShet+ participants. However, these differences were not statistically significant.

#### 3.1.3 Klotho concentrations in the clinical groups

**Figure 1:**
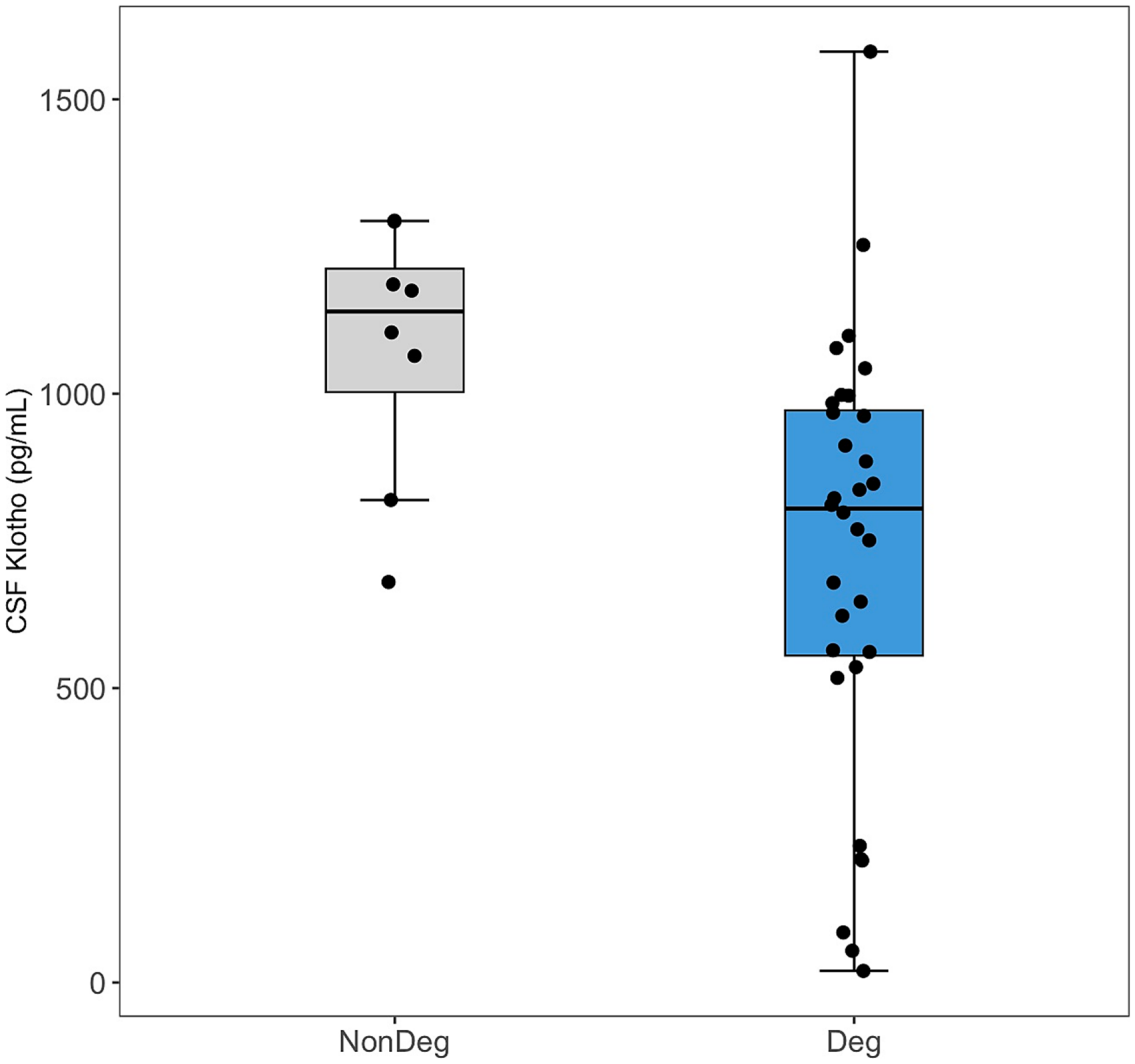
Comparison of Klotho levels in cerebrospinal fluid (CSF) between neurodegenerative (Deg, blue) and non-neurodegenerative (NonDeg, gray) groups. Box plots show the median and interquartile range, with individual data points shown as jittered dots.

CSF Klotho levels were significantly lower in the Deg patients compared to the NonDeg participants (mean = 729 pg./mL, SD = 364 vs. mean = 1,077 pg./mL, SD = 220, *t* = 3.44, *p* = 0.003) (Figure 1). No significant differences in serum Klotho concentrations were observed between the Deg and NonDeg groups.

#### 3.1.4 Association of Klotho concentrations with MMSE scores

CSF Klotho levels, but not serum levels, were positively correlated with MMSE scores in univariate analysis (rho = 0.35, *p* = 0.029) (Table 2).

#### 3.1.5 Association of Klotho concentrations with CP volume

CSF Klotho levels showed a significant negative association with CPVF (rho = −0.32, *p* = 0.042) in univariate analysis (Table 2), whereas no association was found between serum Klotho levels and CPVF. The association was stronger in the Deg subgroup (rho −0.50, *p* 0.004).

### 3.2 Multivariate analysis

#### 3.2.1 CSF klotho model

**Figure 2:**
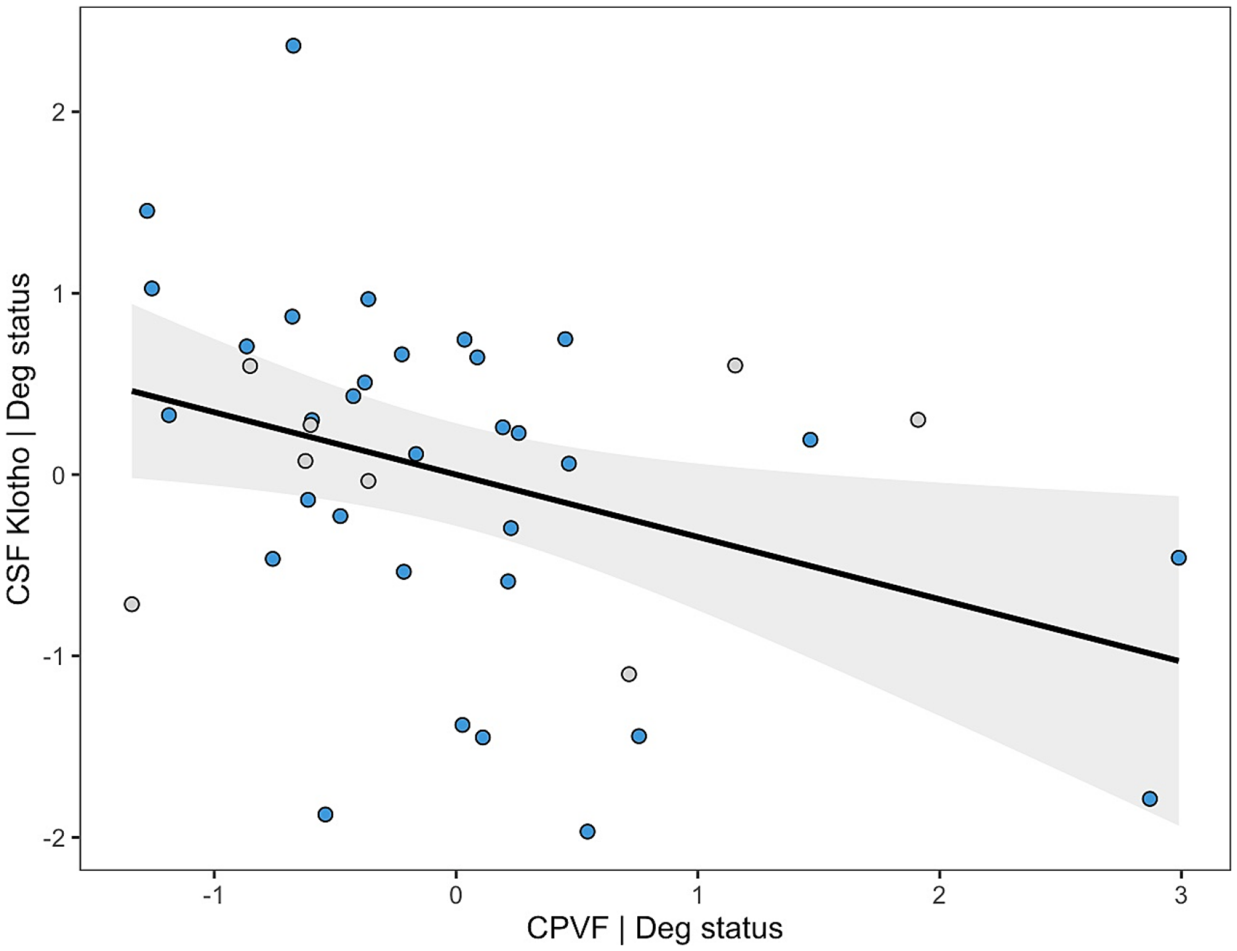
Added variable plot (AVP) showing the relationship between Klotho levels in cerebrospinal fluid (CSF) adjusted for diagnostic group on the y-axis and choroid plexus volume fraction (CPVF) adjusted for the same variable on the x-axis. Blue dots correspond to neurodegenerative patients, and gray dots represent non-neurodegenerative individuals.

The final multivariate linear regression model for CSF Klotho after stepwise variable selection included CPVF and Deg status as predictors, explaining 22.5% of the variance (Adjusted *R*^2^ = 0.225, *F* = 6.54, *p* = 0.004). Deg status (*β* = −1.07, 95% CI [−1.79,−0.35], *p* = 0.005) and higher CPVF (β = −0.34, 95% CI [−0.64,−0.05], *p* = 0.023) were independently associated with lower CSF Klotho levels (Supplementary Table 1; Figure 2).

Deg status (87.4%) and CPVF (76.4%) were consistently retained across bootstrap resamples, indicating high model stability.

In the Deg subgroup, CPVF was retained as the only significant predictor of CSF Klotho levels (β = −0.47, 95% CI [−0.82,−0.13], *p* = 0.009), explaining 18.1% of its variance (Adjusted *R*^2^ = 0.181, *F* = 7.89, *p* = 0.009) (Supplementary Table 2, Supplementary Figure 2).

In the Deg subgroup, CPVF (91.5%) was the only predictor consistently retained across bootstrap iterations.

#### 3.2.2 MMSE model

The final regression model for MMSE scores included Deg status, GMVF, education, and age as predictors, explaining 27.0% of the variance (Adjusted *R*^2^ = 0.270, *F* = 4.60, *p* = 0.004). CSF Klotho levels were not retained as a significant predictor in the model (Supplementary Table 3).

## 4 Discussion

In this preliminary study, we found that CSF Klotho levels were reduced in the neurodegenerative patients compared to the cognitively unimpaired older participants and were inversely associated with CP enlargement on MRI, a putative marker of CP dysfunction.

Moreover, CSF Klotho levels, but not serum levels, were correlated with cognitive scores, although this relationship was evident only before adjusting for covariates.

In our cohort, CSF Klotho concentrations were significantly lower in the patients with AD or bvFTD compared to the cognitively normal older adults. This finding aligns with the only two prior studies that examined CSF Klotho levels in patients with AD, both of which reported similar reductions relative to controls. The absolute CSF Klotho values observed in our cohort are consistent with those reported in the previous two studies (Semba et al., 2014; Grøntvedt et al., 2022). Furthermore, a comparable decrease in CSF Klotho levels has been documented in patients with PD relative to controls (Zimmermann et al., 2021, 2024).

Collectively, these findings suggest that reduced CSF Klotho levels may reflect neurodegenerative processes occurring in the CNS.

In contrast, we did not observe any significant differences in serum Klotho levels between the patients with neurodegenerative conditions and the control participants. The existing literature on this topic is limited and inconsistent. For instance, one study in patients with AD reported no differences in serum Klotho levels compared to controls (Grøntvedt et al., 2022), whereas another study in patients with PD found lower levels in patients versus controls (Yalcin et al., 2024).

Moreover, CSF and serum Klotho levels were not correlated in our cohort. Again, available data are conflicting, with some studies showing that CSF and serum Klotho levels are unrelated or associated only in clinical subgroups (Grøntvedt et al., 2022), others reporting an opposite relationship (Sancesario et al., 2021), and yet another describing a strong relationship between Klotho levels in blood and CSF (Kundu et al., 2022).

Although the lack of association in our results may partly reflect the small sample size and missing serum Klotho data in some patients, the overall absence of a robust relationship supports the notion that Klotho production in CSF and blood occurs independently and is subject to different regulatory mechanisms. In fact, the kidney is the primary source of circulating Klotho, whereas the CP secretes Klotho into CSF.

Consistent with this, we found that serum Klotho levels were predominantly influenced by age and renal function. In fact, serum concentrations decline with advancing age and are inversely associated with markers of renal function, even in normal aging populations (Kuro-o, 2019; Chen et al., 2023; Konnur et al., 2024). Conversely, CSF Klotho levels appear to be less affected by peripheral factors, suggesting that it may serve as a more reliable marker of CNS-specific processes and neurodegeneration (Grøntvedt et al., 2022).

Klotho levels are also influenced by genetic variants, particularly the presence of the KL-VS haplotype in heterozygosity. In our cohort, we observed a noticeable, although not statistically significant, trend toward higher Klotho levels in the KL-VShet+ individuals. This lack of statistical significance may reflect the limited sample size and power of the study. Nevertheless, the observed trend supports the hypothesis that the presence of one copy of the KL-VS variant confers neuroprotective effects through increased Klotho production or stability. However, the differential expression of the Klotho protein across genetic variant subgroups, especially KL-VS homozygotes, and in various fluid compartments remains to be fully characterized (Gaitán et al., 2022).

Our study further showed an association between CSF Klotho levels and cognitive function, as measured by MMSE scores, in univariate analysis. However, this relationship became non-significant after adjusting for confounders such as education and GMVF, which strongly influence cognition and may have obscured a small but meaningful association between CSF Klotho and cognitive performance. Multiple prior studies have reported a similar positive correlation in patients with neurodegenerative conditions (Semba et al., 2014; Grøntvedt et al., 2022; Kundu et al., 2022), although the effect size was smaller in the only study that conducted a multivariate analysis (Grøntvedt et al., 2022). In addition to the inclusion of covariates, the lack of association in our study may be partially attributable to the relatively mild disease stage in most patients, resulting in a narrow range of MMSE scores. Further research is required to definitively elucidate the specific role and mechanisms of Klotho reduction in cognitive dysfunction associated with neurodegeneration.

The most significant finding of our study was the association between CSF klotho levels and CP enlargement. To the best of our knowledge, this is the first study to evaluate this relationship; therefore, no previous data are available to corroborate or refute our findings. Of note, CPVF accounted for a small proportion of CSF Klotho levels, particularly in the Deg subgroup, suggesting that additional factors likely play a substantial role in determining the concentration of this protein in CSF. Nonetheless, this relationship seems biologically conceivable. In fact, not only is the CP the main source of Klotho in the brain, but reduced Klotho production in the CP has been associated with aging and neuroinflammation, which may exacerbate neurodegeneration (Zhu et al., 2018). The effect of Klotho dysregulation at the level of the CP may impact cognitive function and exacerbate neurodegenerative processes through a variety of mechanisms, which include protecting myelin integrity and preventing myelin degeneration in the aging brain (Chen et al., 2013), promoting synaptic plasticity and calcium regulation in the brain (Imura et al., 2007; Chen et al., 2023), modulating neuroinflammation (Sadanandan et al., 2024), and regulating glymphatic clearance (Li et al., 2023). If our results are confirmed, CSF Klotho levels may serve as a novel biomarker of CP enlargement, which could reflect underlying dysfunction in the context of neurodegeneration.

The primary limitation of our study is the relatively small sample size, reflecting both the need for lumbar puncture to obtain CSF samples and the exploratory nature of the research. Owing to its retrospective design, serum Klotho measurements were available only for a subset of participants, as genotyping was prioritized, and Klotho quantification was performed only when sufficient residual blood was available. Moreover, selection bias cannot be ruled out, since only patients with milder clinical presentations typically undergo invasive procedures such as lumbar puncture for CSF collection. Nevertheless, the effect sizes of the main findings are substantial, supporting our conclusions. Nonetheless, we consider our results preliminary, urging readers to interpret our findings with caution and emphasizing the need for replication in larger cohorts.

Second, the cross-sectional design of the study precludes any inference regarding the temporal or causal relationship between reduced CSF Klotho levels and neurodegeneration.

Third, additional MRI sequences (e.g., susceptibility/QSM, FLAIR, or T2-weighted images) were not consistently available across participants, preventing a direct assessment of CP tissue composition. Nevertheless, CP composition may prove more relevant than CP volume itself for understanding plexus dysfunction (Butler et al., 2023). Future studies should therefore include these sequences to better distinguish parenchymal, cystic, and calcified components.

Fourth, cognitive assessment was limited to the MMSE, which may not be sensitive enough to detect subtle or specific cognitive deficits. This limitation could have reduced our ability to uncover associations between reduced Klotho levels and cognitive function. We aim to include larger samples and more detailed cognitive assessments in future confirmatory studies.

In conclusion, in this preliminary study, we showed that Klotho levels are reduced in the CSF of neurodegenerative patients, are associated with CP enlargement, and may be related to cognitive function.

These findings suggest that Klotho could be an indicator not only of brain aging and neurodegeneration but, more specifically, of CP changes in this context. The mechanisms linking Klotho dysregulation in the CP to neurodegeneration, as well as the potential role of Klotho as a biomarker of CP dysfunction, need to be further explored.

## Data Availability

The raw data supporting the conclusions of this article will be made available by the authors, without undue reservation.

## Footnotes

1
http://www.itksnap.org
