# Supplementary material for: Association between Klotho levels in cerebrospinal fluid and choroid plexus enlargement in neurodegeneration
Source: Front Aging Neurosci. 2025 Nov 27;17:1688996. doi: 10.3389/fnagi.2025.1688996 (PMC12695769; doi:10.3389/fnagi.2025.1688996)
Supplement: Supplementary file 1 [file Data_Sheet_1.docx]

***Supplementary table 1****. Results of multivariate linear regression models for Klotho levels in the cerebrospinal fluid (CSF) after forward stepwise selection of variables based on AIC criterion. The table includes regression estimates, standard errors, 95% confidence intervals (CI), t-values, and p-values for each predictor.*

|  | **CSF Klotho** | | | | |
| --- | --- | --- | --- | --- | --- |
| ***Predictor*** | ***Estimate*** | ***Std. Error*** | ***95% CI*** | ***t value*** | ***p-value*** |
| (Intercept) | 0.83 | 0.32 | 0.18,1.47 | 2.59 | **0.014*** |
| CPVF | -0.34 | 0.14 | -0.64,-0.05 | -2.38 | **0.023*** |
| Deg group | -1.07 | 0.36 | -1.79,-0.35 | -3.00 | **0.005**** |
| R^2^ / R^2^ adjusted | 0.26 / 0.22 | | | | |

*Legend: CSF – cerebrospinal fluid; Deg – degenerative group; CPVF – choroid plexus volume fraction*

***Supplementary table 2****. Results of multivariate linear regression models for Klotho levels in the cerebrospinal fluid (CSF) after forward stepwise selection of variables based on AIC criterion in the degenerative subgroup. The table includes regression estimates, standard errors, 95% confidence intervals (CI), t-values, and p-values for each predictor.*

|  | **CSF Klotho** | | | | |
| --- | --- | --- | --- | --- | --- |
| ***Predictor*** | ***Estimate*** | ***Std. Error*** | ***95% CI*** | ***t value*** | ***p-value*** |
| (Intercept) | -0.25 | 0.16 | -0.58,0.08 | -1.55 | 0.13 |
| CPVF | -0.47 | 0.17 | -0.82,-0.13 | -2.81 | **0.009**** |
| R^2^ / R^2^ adjusted | 0.21 / 0.18 | | | | |

*Legend: CSF – cerebrospinal fluid; CPVF – choroid plexus volume fraction;*

2.2 MMSE model

The final regression model for MMSE included Deg status, GMVF, education and age as predictors, explaining 27.0% of the variance (Adjusted R² = 0.270, F = 4.60, p = 0.004). CSF Klotho levels were not retained as a significant predictor in the model (**Supplementary table 3**).

***Supplementary table 3****. Results of multivariate linear regression models for Mini-Mental State Examination (MMSE) scores after forward stepwise selection of variables based on AIC criterion. The table includes regression estimates, standard errors, 95% confidence intervals (CI), t-values, and p-values for each predictor.*

|  | **MMSE** | | | | |
| --- | --- | --- | --- | --- | --- |
| ***Predictor*** | ***Estimate*** | ***Std. Error*** | ***95% CI*** | ***t value*** | ***p-value*** |
| (Intercept) | -0.68 | 0.31 | - | -2.21 | **0.034*** |
| Deg group | 0.77 | 0.34 |  | 2.23 | **0.032*** |
| Education | -0.29 | 0.15 |  | -1.82 | 0.08 |
| GMVF | -0.32 | 0.15 |  | -2.23 | **0.032*** |
| Age | -0.26 | 0.15 |  | -1.68 | 0.10 |
| R^2^ / R^2^ adjusted | 0.35 / 0.27 | | | | |

*Legend: MMSE – Mini-Mental State Examination; Deg – degenerative group; GMVF – grey matter volume fraction*
