# Supplementary figures and images for: Association between Klotho levels in cerebrospinal fluid and choroid plexus enlargement in neurodegeneration
Source: Front Aging Neurosci. 2025 Nov 27;17:1688996. doi: 10.3389/fnagi.2025.1688996 (PMC12695769; doi:10.3389/fnagi.2025.1688996)

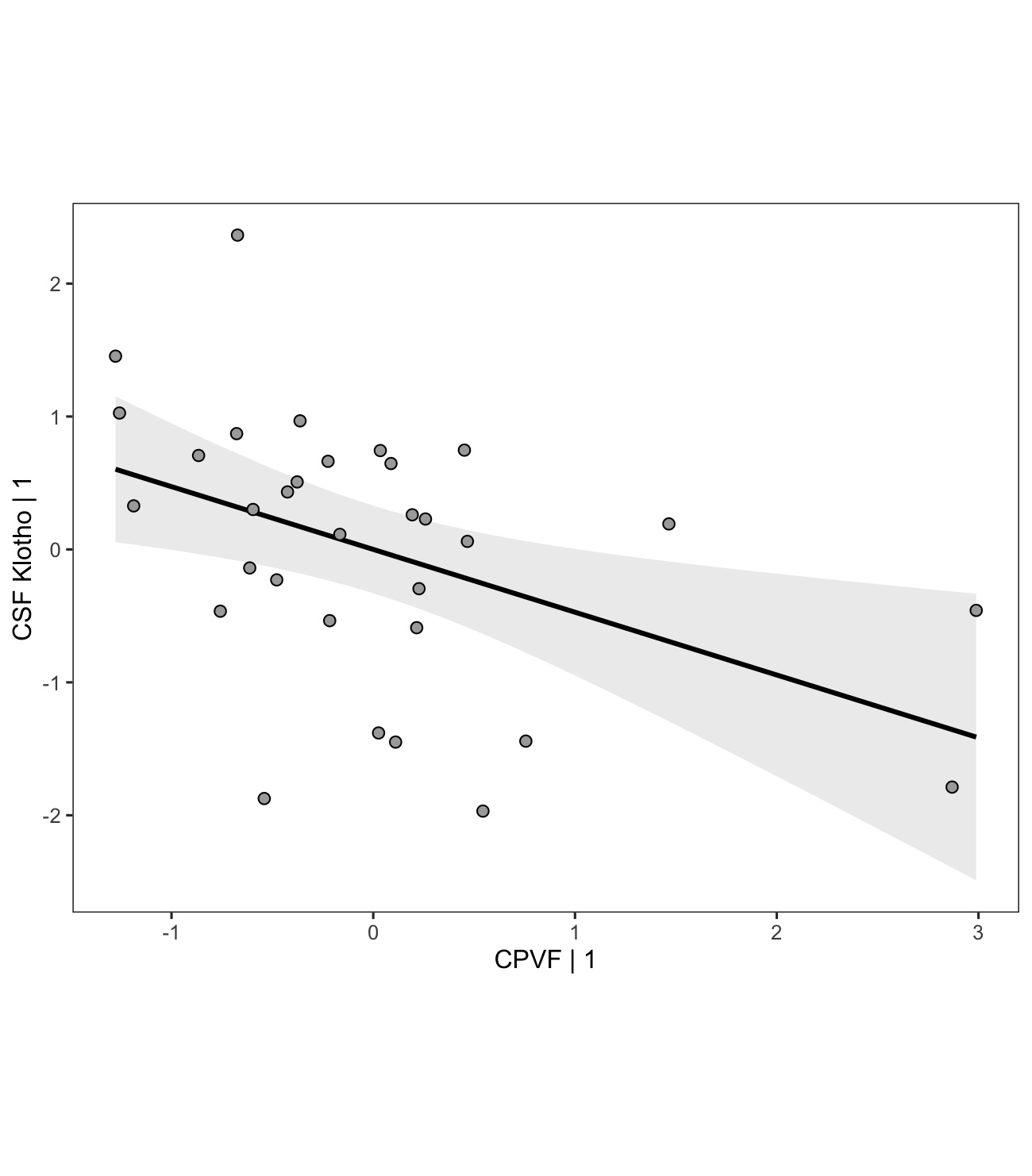

Supplement: Supplementary file 2 [file Image_1.jpeg]
